# An explanatory study of factors influencing engagement in AI education at the K-12 Level: an extension of the classic TAM model

**DOI:** 10.1038/s41598-024-64363-3

**Published:** 2024-06-17

**Authors:** Wei Li, Xiaolin Zhang, Jing Li, Xiao Yang, Dong Li, Yantong Liu

**Affiliations:** 1 Department of Computer Information Engineering, Kunsan National University, Gunsan, 54150 Republic of Korea; 2 Department of Smart Experience Design, Kookmin University, Seoul, 02707 Republic of Korea; 3 Department of Educational Psychology, University of Georgia, Athens, GA 30605 USA; 4 Department of Poultry Science, University of Georgia, Athens, GA 30605 USA; 5 Department of International Culture Education, Chodang University, Muan, 58530 Republic of Korea; 6 College of Art and Design, Guangdong University of Technology, Guangzhou, 510006 China

**Keywords:** AI4k12, GenAI, AIGC, TAM model, Cognitive factors of learning, HCI factors, Artificial intelligence, Psychology, Psychology and behaviour, Computer science, Scientific data

## Abstract

Artificial intelligence (AI) holds immense promise for K-12 education, yet understanding the factors influencing students’ engagement with AI courses remains a challenge. This study addresses this gap by extending the technology acceptance model (TAM) to incorporate cognitive factors such as AI intrinsic motivation (AIIM), AI readiness (AIRD), AI confidence (AICF), and AI anxiety (AIAX), alongside human–computer interaction (HCI) elements like user interface (UI), content (C), and learner-interface interactivity (LINT) in the context of using generative AI (GenAI) tools. By including these factors, an expanded model is presented to capture the complexity of student engagement with AI education. To validate the model, 210 Chinese students spanning grades K7 to K9 participated in a 1 month artificial intelligence course. Survey data and structural equation modeling reveal significant relationships between cognitive and HCI factors and perceived usefulness (PU) and ease of use (PEOU). Specifically, AIIM, AIRD, AICF, UI, C, and LINT positively influence PU and PEOU, while AIAX negatively affects both. Furthermore, PU and PEOU significantly predict students’ attitudes toward AI curriculum learning. These findings underscore the importance of considering cognitive and HCI factors in the design and implementation of AI education initiatives. By providing a theoretical foundation and practical insights, this study informs curriculum development and aids educational institutions and businesses in evaluating and optimizing AI4K12 curriculum design and implementation strategies.

## Introduction

Artificial intelligence (AI) technologies, including blockchain, augmented reality, 3D printing, nanotechnology, and the internet of things, significantly impact various human life aspects^1^. AI’s promise to revolutionize education is evident, with countries like the United States and China actively promoting AI in K-12 education^2–4^. In May 2018, the association for the promotion of artificial intelligence (AAAI) and the association of computer science teachers (CSTA) formed a joint working group to develop national guidelines for K-12 AI education, establishing the K-12 AI education concept (AI4K12)^5,6^. A key advancement in AI research is generative AI (GenAI), which uses machine learning and deep learning to create new data^7–9^. GenAI applications include image generation, natural language processing, and music composition, with innovations like midjourney generated images and ChatGPT smart chat enhancing creativity and public engagement^10–12^. The rise of tools like ChatGPT has intensified GenAI’s role in educational research, drawing public and academic interest to its educational implications, challenges, and opportunities^13–15^.

Research on artificial intelligence in education (AIED) has examined learners’ receptivity, Technological, system quality, cultural, self-efficacy, and trust factors are deemed crucial in e-learning systems^10–12^. Studies in computer vision courses highlight the influence of prior knowledge, skills, learning styles, motivation, and self-efficacy, the usability of the system, observable rows, and experimentation also affect the use of computer tools in the classroom^16–19^. Students’ perspectives on employing ChatGPT in programming and programming education^20^. A scale was developed, based on the unified theory of acceptance and use of technology (UTAUT) model, to gauge students’ acceptance of AI applications generated by artificial intelligence^21^. This scale was tailored and crafted for individuals aged 18–60 in Turkey. The validity and reliability of the AI literacy scale were confirmed^22^. Studies on the utilization of chatbots in training programs disclose that social expectations, effort, and influence are pivotal factors for engagement^23^.Chai et al. explored the correlation between AI literacy, AI curriculum framework (AICF), social welfare, and behavioral intention (BI) in K-12 students, finding positive correlations among these elements^24^. Long and Magerko (2020) developed a framework for AI literacy in K-12, emphasizing design considerations like explainability and transparency^25^. Green et al.(2019) proposed disciplinary literacy instruction in K-12 engineering to address diversity barriers in engineering careers^26^. However, few studies have systematically examined K-12 students’ acceptance of AI programs, particularly the causal relationships and direct impact factors. Students’ perceptions, cognitive factors in AI learning^27^, GenAI tools’ interactivity, and human–computer interaction (HCI)^28^ factors are all crucial in influencing acceptance of AI in education. Studying K-12 students’ attitudes towards AI courses using GenAI tools is a promising research area, vital for understanding engagement, learning outcomes, and course optimization^29^.

In this study, we adopt the technology acceptance model (TAM) as the theoretical framework to understand K-12 students’ attitudes towards AI courses using generative AI (GenAI) tools. TAM, has been widely utilized to assess users’ acceptance and adoption of new technologies. The model posits that perceived usefulness (PU) and perceived ease of use (PEOU) significantly influence users’ attitudes and behavioral intentions towards adopting a new technology. Building upon this foundation, our extended TAM incorporates cognitive factors related to AI learning, such as AI intrinsic motivation (AIIM), AI readiness (AIRD), AI confidence (AICF), and AI anxiety (AIAX), alongside human–computer interaction (HCI) elements like user interface (UI), content (C), and learner interface interactivity (LINT) specific to GenAI tools. By integrating these additional constructs into the TAM framework, we aim to provide a more comprehensive understanding of the factors shaping K-12 students’ acceptance of AI courses facilitated by GenAI tools.

Recent empirical studies have shed light on various aspects of AI in education, providing valuable insights into factors influencing students’ attitudes and behaviors. For instance, research by Almaiah and Almulhem (2018) identified key success factors for e-learning system implementation using the Delphi technique^10^. Similarly, Almaiah, Al-Khasawneh, and Althunibat (2020) explored critical challenges and factors influencing e-learning system usage during the COVID-19 pandemic^11^. Thematic analysis by Almaiah and Al Mulhem (2020) classified main challenges and factors influencing the successful implementation of e-learning systems using NVivo^12^. These studies underscore the importance of understanding the dynamics of technology acceptance and usage in educational settings, providing valuable insights that inform our research approach and contribute to the broader discourse on AI in education.

This study aims to analyze K-12 students’ attitudes towards AI courses using GenAI tools. Employing a conceptual model based on the technology adoption model (TAM), it includes cognitive and HCI factors as external variables in an extended model. The study involves designing and implementing GenAI-based AI courses. A group of 210 Chinese K7–K9 students participated, with their experiences evaluated through post-course questionnaires. Hypotheses were tested using structural equation modeling, resulting in an enhanced TAM version.

The study’s innovative contributions are:

1. Developing a comprehensive set of indicators for factors influencing K-12 students’ attitudes towards GenAI tool-based courses.
2. Experimentally deriving interrelationships among these influencing factors.
3. Proposing an improved experimental methodology based on the TAM model to validate these relationships.

As K-12 AI education advances, the significance and refinement of related models and frameworks are expected to grow.

This study proposes an extended TAM combining students’ cognitive learning of AI courses with GenAI’s HCI factors, potentially offering new directions for TAM in GenAI-based education in K-12.

## Literature review and hypothesis

Artificial intelligence (AI) is increasingly pivotal in education^30^. AI in education (AI-Ed) involves computers executing cognitive tasks akin to human thinking, particularly in learning and problem-solving. Over the past 30 years, AI-Ed has integrated into the education sector through various means, the integration of intelligent educational methods, curriculum design, and course structure aims to imbue students with environmental and sustainable development (ESD) awareness, while simultaneously incorporating cutting-edge technologies like artificial intelligence within the ESD framework^31^. This integration includes AI monitoring student forums, intelligent assessments, serving as a learning companion, assisting or replacing educators, and functioning as private tutors. Moreover, AI-Ed serves as a research tool for advancing science education^32^. Utilizing AI-Ed in computer science, machine learning, and deep learning can bridge the digital divide and foster AI literacy^13^. Consequently, AI has become an integral subject in K-12 education, preparing students with digital-driven knowledge and problem-solving skills for the digital world^33^.

Generative AI (GenAI) is a subset of AI that has garnered significant attention. It allows users to create new content, including text, images, audio, video, and 3D models, based on input requests. Recently, several GenAI platforms have emerged, such as ChatGPT, a large language model launched on November 30, 2022, which attracted a million users within five days of its release^34,35^. ChatGPT, as an AI chatbot, aids student learning by providing information and narration^36^. In the realm of GenAI imagery, platforms like Disco Diffusion, Dall-E2, Imagen, Mid Journey, and Stable Diffusion are prominent. Mid Journey, for example, creates artistic images based on user text inputs^37^, impacting art and education. These GenAI applications generate outputs after learning from user requests. Applying GenAI to AI4K12 involves interpreting science and technology through engineering and art^38^. For instance, using ChatGPT and Mid Journey in AI education practice for K-12 involves designing courses with visual narratives^39^. ChatGPT enhances students’ communication and narrative skills, while Mid Journey can be used to create picture books^40^. Incorporating tools like ChatGPT and Mid Journey into curriculum design is feasible for improving AI literacy in K-12 education.

Previous research has delved into various facets of AI in Education (AI-Ed), exploring educators’ readiness to teach AI, attitudes towards using chatbots in education, and factors influencing students' continued interest in AI learning. However, despite these valuable insights, there remains a gap in understanding the factors specifically influencing K-12 students’ acceptance of AI courses facilitated by generative AI (GenAI) tools. Given the increasing integration of AI into K-12 education and the emergence of GenAI platforms, it is crucial to explore the unique dynamics shaping students’ attitudes towards these innovative learning tools. By addressing this research gap, our study aims to contribute to the existing literature by providing insights into the factors driving K-12 students’ acceptance of AI4K12 courses, ultimately informing the design and implementation of effective AI education programs for this demographic.

Previous studies have examined various perspectives in AIED. These include educators’ readiness and willingness to teach AI^18^, combining diffusion theory with technology adoption rates confirms that usability and user-friendliness are relevant to the adoption rate of artificial intelligence tools in online learning^19^, and attitudes towards using chatbots in education^41^. Research has also focused on factors influencing students’ continued interest in AI learning^24^, perceptions of AI coaching^23^, and universities’ behavioral intention (BI) to use AI robots for education^42^. To foster the widespread adoption of GenAI programs in AI4K12, understanding the factors influencing K-12 students’ acceptance of such courses is essential. This study aims to explore these influential factors.

The technology acceptance model (TAM), originally proposed by Davis, provides a robust theoretical framework for examining user acceptance and usage of new technologies. While TAM has been widely applied across various fields, including healthcare, management, and finance, its application in the context of AI in education (AIED) remains relatively underexplored. Specifically, the unique characteristics of GenAI tools and their implications for students’ perceptions of usefulness and ease of use have not been thoroughly investigated within the TAM framework. By applying TAM to the study of K-12 students’ attitudes towards AI courses with GenAI tools, our research seeks to elucidate the underlying factors driving students’ acceptance of these innovative learning platforms. This theoretical approach allows us to identify key determinants of students’ attitudes and intentions towards AI4K12 courses, providing valuable insights for educators, policymakers, and developers seeking to enhance AI literacy and engagement among K-12 students.

The technology acceptance model (TAM), proposed by Davis, addresses user acceptance and usage of new technologies^43,44^.

Based on TAM model, this paper explores the willingness of university students to use the meta-universe-based learning platform. Perceived usefulness, personal innovation and perceived enjoyment are the key factors^45^. He suggested that perceived usefulness (PU) and perceived ease of use (PEOU) are key to embracing and promoting technology use^43,44^. TAM has been applied across various fields, including healthcare, management, finance, and education, For example: the university student to the mobile learning acceptance degree research^46,47^. In AI device acceptance studies, a theoretical model called AI device usage acceptance (AIDUA) includes social influence, personification, performance expectations, emotional engagement, and hedonic motivation as antecedents to user attitudes^48^. Other studies on AI acceptance have identified PU, performance expectations, attitude, trust, and effort expectation as influencing AI intention, willingness, and usage behavior^49^. Research on students’ willingness to continue AI learning revealed that AI literacy and its impact on social welfare affect students’ BI^24^. Applying TAM in AIED shows varying external variables influencing PU and PEOU from different research angles. The factors influencing K-12 students’ attitudes towards learning AI courses with GenAI tools remain unclear.

## Materials and methods

### External variables

#### Learning cognition of AI

Based on the cognitive characteristics of students in AI education for K-12 (AI4K-12), we propose the inclusion of four key variables to enhance the technology acceptance model (TAM) study: AI intrinsic motivation (AIIM), AI readiness (AIRD), AI confidence (AICF), and AI anxiety (AIAX).

This suggestion stems from a comprehensive review of teachers’ AI cognition and their willingness to teach AI, highlighting the importance of understanding AIAX, its impact on social welfare, attitude towards use (ATT) AI, perceived teaching confidence, AICF, AI correlations, AIRD, and behavioral intentions (BI). Furthermore, in the context of AI4K12, students’ cognition plays a crucial role in influencing their learning process and outcomes. Our research on developing and evaluating AI courses confirms the significance of learning perception abilities, including motivation, confidence, attitude, readiness, and anxiety, in shaping effective AI education strategies.

AI intrinsic motivation (AIIM): Previous study has shown that motivation can enhance students’ willingness to learn^50–52^. Intrinsic motivation possesses a psychological cognitive process of exploration, experimentation, curiosity, and manipulation, which is a natural manifestation of human learning and integration of knowledge^53^. The intrinsic motivation of learning guides students to set learning goals and continuously participates in the learning process through the classroom learning activities, which has a positive impact on academic performance^54^. Therefore we propose the following assumptions.

- H1a, students’ AIIM has a positive impact on their PU in learning AI courses through the GenAI tool.
- H1b, students’ AIIM has a positive impact on their PEOU in learning AI courses through the GenAI tool.

AI readiness (AIRD): the technology readiness index (TRI) is used to measure people’s tendency to accept and use advanced information technology^55^. Based on positive expectations for the use of technology, preparatory work can predict learning behavior^56^. AIRD can measure students’ understanding of the comfort level of AI knowledge and technology in their learning and life, and has a related impact on their learning attitude towards AI courses^57^. In the behavioral research of teachers teaching AIED, AIRD is related to BI, and PU has a positive impact on BI. Therefore, we propose the following hypothesis.

iii. H2a, students’AIRD has a positive impact on their PU in learning AI courses through the GenAI tool.
iv. H2b, students’AIRD has a positive impact on their PEOU in learning AI courses through the GenAI tool.

AI confidence: in AIED, AICF represents students’ confidence in learning AI course content^58^. AICF can affect students’ willingness to learn and other variables, and is an important impact factor on AI usage behavior^59–61^. In research on students using mobile devices for learning, it has been found that mobile device usage confidence has a positive impact on PEOU^62,63^. Therefore, we propose the following assumptions.

e. H3a, students’ AICF has a positive impact on their PU in learning AI courses through the GenAI tool.
f. H3b, students’ AICF has a positive impact on their PEOU in learning AI courses through the GenAI tool.

AI anxiety: computer phobia is defined as the fear and anxiety of advanced technology^64^. When using mobile devices for learning, mobile device anxiety can also affect learning behavior^63^. Based on the background of AI, AIAX can be traced back to technology phobia and computer anxiety. Define AIAX as a fear of AI, and users' concerns about the unknown impact of AI programs and related technological developments on humans and society^65,66^. In the use of ChatGPT, AIAX predicts learning behavior^67,68^, and the unease of GenAI usage affects user behavior^69,70^. In e-learning environments, where learners interact with AI tools during the process, anxiety and uneasiness affect the user’s usage.AIAX has an impact on PU^71^. In using the GenAI tool to learn AI courses, we propose the following assumptions.

g. H4a, students’ AIAX has a negative impact on their PU in learning AI courses through the GenAI tool.
h. H4b, students’ AIAX has a negative impact on their PEOU in learning AI courses through the GenAI tool.

#### HCI factors in AIED

HCI refers to the interaction between users and computers. And human–computer interaction refers to the computer-mediated dialogue that users engage in the created environment by themselves. Interactivity in online educational programs refers to the relationship between students and computers in a human–computer interaction environment^72^. During the process of using the GenAI tool for AIED, HCI has an impact on students’ attitudes and behaviors^67^. Therefore, based on the teaching characteristics of using the GenAI tool in AI4K12, we suggest that considering HCI factors and using interface design (UI), content (C), and learner interface interactivity (LINT) as variables to expand TAM research.

using interface design (UI): in HCI, an interface is defined as the visible part of the information system that can be touched, heard, and seen by the user^72^. UI is an important factor in the software development process, and user demand oriented design is the key to UI^73^. The emergence of user centered UI principles provides a theoretical basis for designers to conduct UI, such as distinguishing the most important information, buttons with consistent styles, and actively providing feedback^74,75^. In the research field of online courses or mobile applications for learning, following UI principles makes the system easier for students to use and operate, and UI also plays an important role in the system's PU, Based on the technology acceptance model, learning content quality, content design quality, interactivity, functionality, user interface design, accessibility, personalization, and responsiveness are the main factors influencing the acceptance of mobile learning^76,77^. In the process of using GenAI for teaching, the UI also has an impact on PEOU. Therefore, we propose the following assumptions.

i. H5, the UI of the GenAI tool used in AI course learning has a positive impact on PEOU.

Content (C): C is related to the course content. In the field of mobile devices, C is considered to have a significant impact on student satisfaction^78^. In the computer context, the structure and capacity of C have a direct impact on PU, and C is an important influencing factor for user acceptance of the system^79^. When investigating the factors that affect the use of BI on mobile devices, C has a positive impact on PU^63^. In evaluating the role of MOOC acceptance and use, C is positively correlated with PEOU^80^. Based on previous research findings, we propose the following assumptions.

j. H6a, the use of GenAI tools for teaching’s C has a positive impact on students’ PU in learning AI courses.
k. H6b, the use of GenAI tools for teaching's C has a positive impact on students’ PEOU in learning AI courses.

Learner interface interactivity (LINT): LINT allows users to interact with the system through the menu bar using the program^81^. When testing the impact of enhancing student interactivity on improving e-learning acceptance and the relationship between variables, there is a relationship between LINT, PU, and PEOU^29^. During the use of GenAI tools, LINT also has an impact on students’ PU and PEOU, so we assume that.

xii. H7a, LINT has a positive impact on students’ PU in learning AI courses through the GenAI tool.
xiii. H7b, LINT has a positive impact on students’ PEOU in learning AI courses through the GenAI tool.

### Internal variables

Perceived Usefulness (PU) is defined as the degree to which a user believes that using a specific system will improve their/her work performance. In addition, perceived ease of use (PEOU) is defined as the degree to which users do not need to put in any effort to use the system^43,44^. The correlation between TAM model structures has been proven in many studies. The relationship between PU and PEOU has also been confirmed in research in the field of education. Attitude towards use (ATT) is a person’s perception of technology, which is a psychological feedback of liking, enjoying, and being happy with technology^58^, usability, which has a positive impact on the practical use of m-learning systems^82^. In the previous research, there is a higher education students to adopt the meta-educational intention of the factors^83^. and studies on users’ sustained intention towards e-learning^84^ have both concluded that both PU and PEOU affect a person’s ability to use the system’s ATT. Therefore, when studying the influencing factors of students’ attitudes towards AI teaching using GenAI, we propose the following assumptions:

n. H8, the PU of AI courses learned by students through the GenAI tool has a positive impact on ATT.
o. H9, students’ learning of AI courses through the GenAI tool has a positive impact on PU through PEOU.
p. H10, the PEOU of students learning AI courses through the GenAI tool has a positive impact on their attitude towards ATT.

### Model

#### Research model

**Figure 1:**
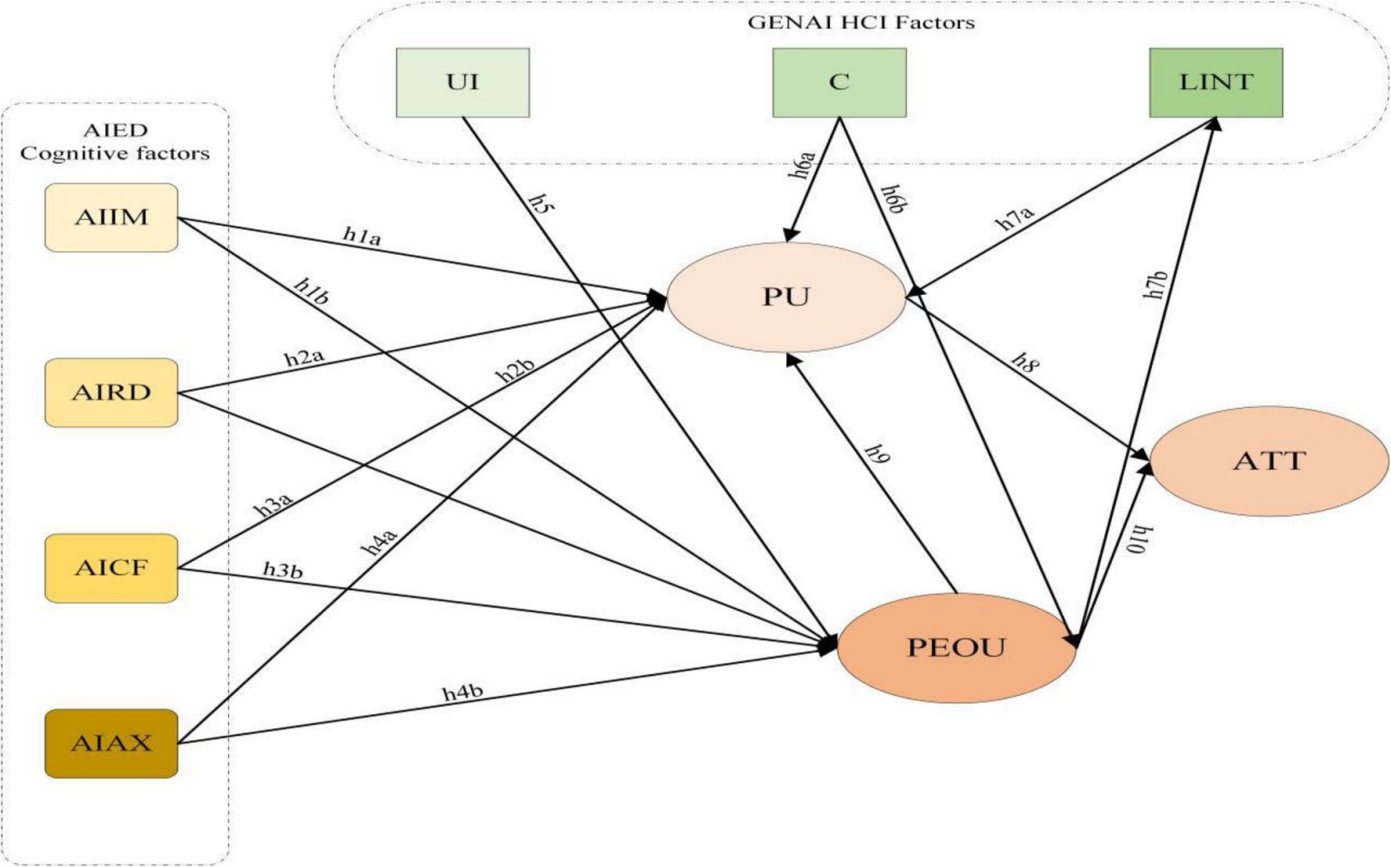
Assumption model.

This study analyzed the learning cognitive and human interaction factors that affect students’ attitudes. Expand Davis’ TAM model with external variables from literature review and previous research findings. Using PU, PEOU, and ATT as basic variables, seven external variables were derived through literature review and previous research analysis. Figure 1 shows the proposed hypothesis model.

This study endeavors to delve into the determinants shaping K-12 students’ perceptions of AI courses facilitated by generative AI (GenAI) tools. To elucidate these factors, an analytical framework was formulated, drawing inspiration from Davis’ technology acceptance model (TAM) as its foundational underpinning. Building upon the core constructs of TAM—perceived usefulness (PU), perceived ease of use (PEOU), and attitude towards use (ATT)—the research extends the model by incorporating additional external variables gleaned from an exhaustive literature review and synthesis of prior research. Specifically, the model integrates cognitive factors associated with AI learning, including AI intrinsic motivation (AIIM), AI readiness (AIRD), AI confidence (AICF), and AI anxiety (AIAX), as well as human–computer interaction (HCI) elements such as user interface (UI), content (C), and Learner Interface Interactivity (LINT). Figure 1 depicts the proposed hypothesis model, illustrating the interconnections among these variables. For participant selection, a convenience sampling approach was adopted to recruit a cohort of 210 Chinese K-12 students spanning grades K7–K9. This sampling method was chosen for its practicality and ease of access, facilitating the efficient enlistment of participants from the target demographic. Demographic details, encompassing age, gender, and grade level, were gathered to furnish insights into the profile of the sample, enabling a more nuanced analysis of the research outcomes.

In terms of tool development and validation, all research instruments utilized in this study were selected or adapted from established measures drawn from prior research endeavors. Rigorous attention was dedicated to ensuring the reliability and validity of these measures, with necessary adjustments made to align them with the study context. Validation procedures encompassed pilot testing and expert validation to affirm the appropriateness of the measures in assessing the intended constructs. Through this meticulous validation process, the research instruments were deemed apt for capturing the pertinent variables of interest.

Data analysis procedures entailed the utilization of structural equation modeling (SEM) techniques to analyze the quantitative data collected through surveys. This analytical approach facilitated the testing of the stipulated hypotheses and the exploration of the relationships between the variables delineated in the research model. Statistical software packages such as SPSS and AMOS were employed to conduct the analyses, enabling robust statistical testing and elucidation of the research findings. By organizing the methodology section in a cohesive narrative format, this study offers a lucid and transparent depiction of the research design, participant recruitment approach, measurement instruments, and data analysis protocols, ensuring rigor and validity in the study’s outcomes.

#### Participants and experimental procedures

**Figure 2:**
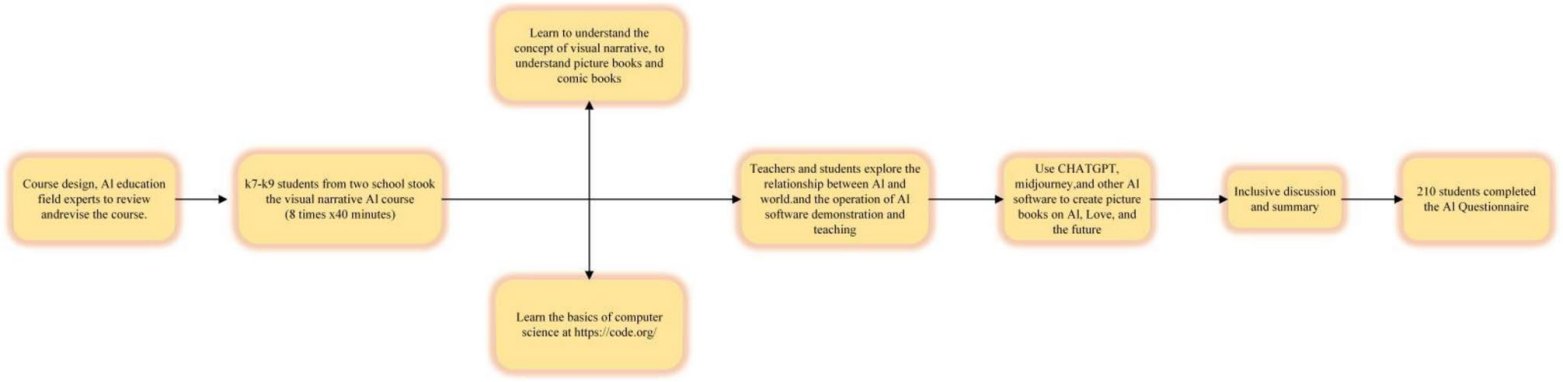
Experimental process.

The participants in this study were 210 students selected from two high schools in China. Among them, 97 were males (45.7%) and 114 were females (54.3%). The students’ grades are K7-K9. Students voluntarily participate in research experiments and are aware of the research procedures. The data related to the experiments are anonymous and have also received permission and recognition from their parents and the school. Students will participate in a one month course, which mainly focuses on AI knowledge learning using the GenAI tool. The main content of the course is the creation of AI visual narratives (story picture books). All students are undergoing systematic AIED for the first time. The experimental process is shown in Fig. 2.

Sample population: the sample population for this study consisted of K-12 students from various schools in China. These students were chosen to represent a diverse demographic, including different grade levels and socioeconomic backgrounds, to ensure the findings were applicable across a broad range of contexts.

Sampling technique: a stratified random sampling technique was employed to select participants for the study. Schools were stratified based on geographic location, school type (public/private), and grade level. Within each stratum, a random sample of schools was selected, and then students within those schools were randomly chosen to participate in the study. This sampling technique helped ensure that the sample was representative of the target population and minimized selection bias.

Justification of sample size: the sample size of 210 Chinese K-12 students was determined based on power analysis and the requirements for structural equation modeling (SEM) analysis. Prior research suggests that a sample size of at least 200 participants is adequate for SEM analysis, particularly when examining complex relationships among variables. Additionally, power analysis was conducted to ensure that the sample size was sufficient to detect meaningful effects with a reasonable degree of confidence. This sample size also allowed for subgroup analyses based on demographic variables such as grade level and gender, providing further insights into potential variations within the sample population.

#### Experimental implementation and feedback

The course spans one month, comprising a total of eight classes, dedicated to the creation of AI picture books centered on “AI, Love, and the Future”. From sessions 3 to 7, students delve into utilizing ChatGPT, Midjourney, and AI translation software for crafting their picture books. Collaboratively, teachers and students explore the nexus between AI and our world, leveraging GenAI for acquiring novel knowledge. This encompasses mastering AI translation software for bilingual tasks and harnessing generative chat tools for narrative continuity. Additionally, understanding how generative image systems operate in image creation and story coherence is emphasized. The final session involves student presentations, fostering discussions and idea exchanges between teachers and students. Course content design adheres to input from five AIED experts, detailed in Table 1.

**Table 1:** Course design.

Course	Activities
1–2	Understand and learn the concept of visual narrative, have a specific understanding of picture books and comic strips, and explain the theme of this activity as AI, future life, and an equal world
	What is AI?
	A brief history of AI development
	Learning AI courses via https://code.org/, and the basics of computer science
3–7	Understand and initially use AI tools in different fields, especially the intelligent chat software ChatGPT, AI painting software Midjourney, and online translation software
	Teachers and students jointly explore the relationship between AI and us, and in what aspects will AI assist in intervening in our lives now and in the future? Will AI cause inequality?
	The teacher demonstrates how to use the above tools to create a picture book with their own theme
	Students try to determine their own picture book themes through communication, and then use intelligent search engines, ChatGPT, and Midjourney to create picture books that match text. When encountering problems during the creative process, students and teachers will also share and communicate with each other
8	After the completion of the work, the teacher communicates with the students and explores the gains and problems discovered during the course learning process
	Finally, complete the course feedback and students will fill out the AI questionnaire

**Figure 3:**
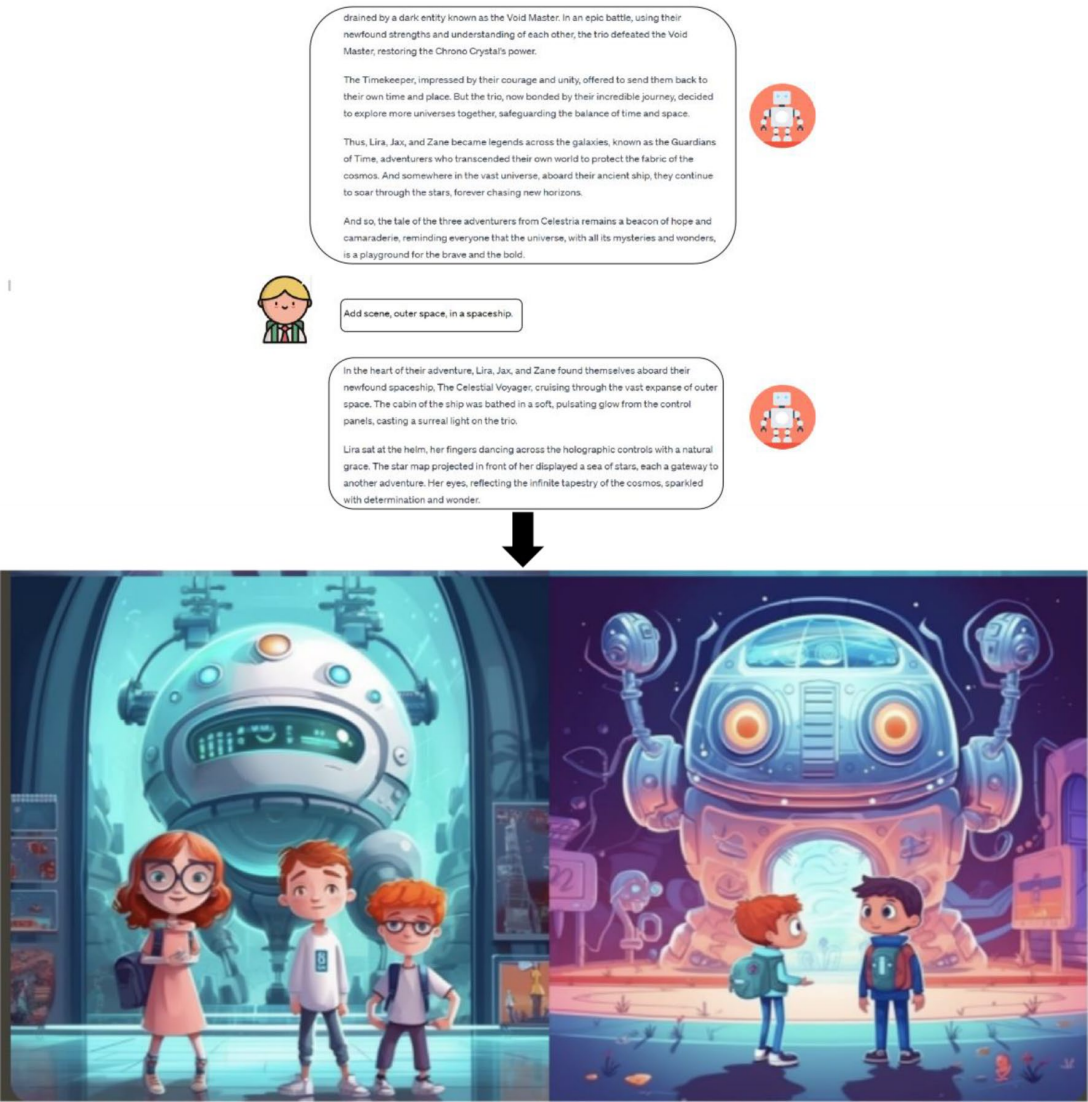
Course implementation process.

During the course implementation process, students use ChatGPT and Midjourney to create and showcase their works, as shown in Fig. 3 of the course implementation process.

### Questionnaire design

The survey instrument is divided into two parts. The first part of the survey questionnaire includes demographic questions, including gender, grade; and the second step uses 38 items to measure the 10 structures of the research model. Ten structures are classified as external variables and internal variables.

- External variables (AIIM, AIRD, AICF, AIAX, UI, C, LINT).
- Internal variables (PU, PEOU, ATT). Each construct is measured by multiple items. In order to obtain participants’ responses and quantify the construction, a five-point Likert scale was used to score the questionnaire responses. The Likert scale consists of five answer options, ranging from “strongly disagree” (mapped to number 1) to “strongly agree” (mapped to number 5).

This tool was developed after reviewing research on TAM models, AI learning cognitive factors, and HCI factors. All items in the survey questionnaire were proofread by translation experts and translated into Chinese. The specific content and reference materials of the variable item survey questionnaire are shown in Table 2.

**Table 2:** Specific content and reference materials of the survey questionnaire.

Variables	Items	Reference
Perceived usefulness (PU) (4 items)	PU1, Using GenAI tools can help me learn more about artificial intelligence (AI) knowledge	Davis.(1989)^44^, Lee.(2010)^84^
	PU2, Using GenAI tools in PU2 can improve the efficiency of my learning of AI courses	
	PU3, Using GenAI tools will help me complete learning tasks faster	
	PU4 Using GenAI tools will make learning AI knowledge easier for me	
Perceived ease of use (PEOU) (4 items)	PEOU1, It is easy for me to learn AI courses using the GenAI	Davis.(1989)^44^, Lee.(2010)^84^
	PEOU2, I found that my interaction with GenAI tools does not require too much mental effort on my part	
	PEOU3, I found it easy to have GenAI tools do what I want them to do	
	PEOU4, I found GenAI tools easy to use	
Attitudes towards the use of artificial intelligence (ATT) (4 items)	ATT1, I think learning AI courses through GenAI has given me great satisfaction	Xia et al. (2011)^26^, Davis.(1989)^44^, Lee.(2010)^84^
	ATT2, I think learning artificial intelligence courses through GenAI is an interesting thing	
	ATT3, I believe that GenAI is a valuable tool for learning AI courses	
	ATT4, I believe that learning AI courses through GenAI tools has given me a unique experience	
Student’s intrinsic motivation to learn Artificial intelligence (AIIM) (4 items)	AIIM1, In this course of learning AI through GenAI, I prefer AI topics that can arouse my curiosity, even if they are difficult to learn	Chiu et al. (2011)^27^, Duncan & Mckeachie(2005)^85^
	AIIM2, In this course on learning AI through GenAI, I prefer materials that truly challenge me, so that I can learn new things	
	AIIM3, What satisfies me the most about learning AI through GenAI is understanding the content as thoroughly as possible	
	AIIM4, I enjoy learning in this AI course using GenAI tools	
Artificial intelligence readiness (AIRD) (4 items)	AIRD1, AI technology allows people to better control their lives	Xia et al. (2011)^26^, Chiu et al. (2011)^27^
	AIRD2, Products and services that use the latest AI technology are more convenient to use	
	AIRD3, I prefer to use the most advanced AI technology	
	AIRD4, I like AI technology, which allows me to customize applications to meet my needs	
Artificial intelligence confidence (AICF) (4 items)	AICF1, I am confident in achieving good results in this course of learning AI through GenAI	Xia et al. (2011)^26^, Chiu et al. (2011)^27^
	AICF2, I am confident that if I work hard enough in this course through GenAI tools, I will definitely succeed	
	AICF3, I am confident that I can understand the most difficult material in the course of learning AI knowledge through GenAI	
	AICF4, I am confident that I can learn the basic concepts taught in the course of Learning AI through GenAI	
Artificial intelligence anxiety (AIAX) (4 items)	AIAX1, When I consider the ability to learn AI knowledge through GenAI, I think about how difficult my future will be	Xia et al. (2011)^26^, Wang & Wang (2022)^66^
	AIAX2, When I think of learning AI knowledge through GenAI, I have a feeling of unease and unease	
	AIAX3, Using GenAI as a system is a bit intimidating for me	
	AIAX4, I hesitate to use a GenAI system because I am concerned about making errors that cannot be corrected	
User interface (UI) (3 items)	UI1, The screen design of GenAI tools is very comfortable to use	Nikou & Economides. (2017)^63^, Liu et al.(2010)^76^
	UI2, Browsing GenAI tool pages is very simple	
	UI3, I like the interactivity provided by GenAI tools	
Content (C) (4 items)	C1, The GenAI tools are clear and easy to understand	Nikou & Economides. (2017)^63^, Terzis & Economides.(2011)^79^
	C2, The GenAI tools are related to the course syllabus	
	C3, The GenAI tool is very useful for my course	
	C4, The content and presentation methods of GenAI have attracted attention	
Learner-interface interactivity (LINT) (3 items)	LINT1, Access GenAI anytime and use it online	Al-Sayid & Kirkil.(2023)^29^, Chou.(2003)^86^
	LINT2, using GenAI with some online help	
	LINT3, completes AI course tasks faster by using GenAI	

### Questionnaire collection and demography

Following the course completion, students anonymously and voluntarily completed a questionnaire survey. The questionnaire was administered via the Chinese online platform, question star, resulting in 210 responses. Post-sorting, 13 responses were deemed invalid, leaving 197 valid ones. Demographic variables underwent frequency analysis utilizing SPSS 26 software, revealing a distribution of 86 boys (43.7%) and 111 girls (56.3%). Among them, 65 students were in seventh grade (33%), 70 in eighth grade (35.5%), and 62 in ninth grade (31.5%). Demographic information is summarized in Table 3.

**Table 3:** Demographic information of students.

variables	Item	Frequency	Effective percentage	Cumulative percentage
Gender	Male	86	43.7	43.7
	Female	111	56.3	100
Grade	Grade 7	65	33	33
	Grade 8	70	35.5	68.5
	Grade 9	62	31.5	100

All methods were performed in accordance with relevant ethical guidelines and regulations, the experimental protocols were approved by the Academic Committee of Guangzhou University of Technology and Guangzhou University of Technology, and the experiments were conducted with the informed consent of all subjects and their legal guardians.

### Data analysis methods

This study used SPSS 26 and SMART PLS 4.0 for data analysis. Data analysis includes two steps, reliability and validity analysis, as well as hypothesis testing. Firstly, internal consistency reliability (Cronbach's α and composite reliability} was measured by SMART PLS 4.0; and composite reliability (CR) was tested using SPSS 26. High CA and CR values indicate high reliability of the tool. It is recommended that the CA and CR values be higher than 0.70. To evaluate the convergence effectiveness of the construction, we used CR values and average variance extraction (AVE) values, and verified the discriminant effectiveness of the construction by analyzing the square root value of the extracted mean difference (AVE). If all constructs are higher than the correlation between constructs, then the sufficiency of discriminative validity is demonstrated. Secondly, after obtaining satisfactory results in the first step, use the structural model to test the hypothesis. Analyze the significance and magnitude of each path coefficient to test our hypothesis. The model fitting index was also evaluated to determine the adequacy of the proposed research model.

### Experimental bias

To mitigate the potential effects of common method bias, several strategies were employed throughout the data collection and analysis processes. First, we ensured anonymity and confidentiality in the survey responses to encourage participants to provide honest and accurate answers without fear of judgment or repercussion. Additionally, we employed procedural remedies such as counterbalancing the order of questionnaire items and using reverse-coded items to minimize response bias. Furthermore, we conducted Harman’s single-factor test to assess the extent of common method bias in our data. The results indicated that no single factor accounted for the majority of the variance, suggesting that common method bias was not a significant concern in our study. However, we acknowledge that these measures may not completely eliminate common method bias and have included this limitation in our discussion.

## Results

During the course implementation process, students use ChatGPT and Midjourney to create and showcase their works, as shown in Fig. 3 of the course implementation process.

### Evaluation of measurement tools

#### Results of reliability and effectiveness testing

Table 4 shows the reliability analysis results by SPSS 26, and the Clonbachα values meet the standard, all greater than 0.8. Therefore, it can be proven that the research results of variables are reasonable. To ensure the accuracy of measurement results, reliability analysis needs to be conducted on the valid data in the questionnaire before analysis.

**Table 4:** Reliability analysis results.

Dimension	Item	Corrected item and total correlation	Clonbachα after deleting item	Dimension total α
Perceived usefulness	PU1	0.676	0.874	0.882
	PU2	0.788	0.830	
	PU3	0.761	0.841	
	PU4	0.752	0.845	
Perceived ease of use	PEOU1	0.728	0.895	0.904
	PEOU2	0.790	0.873	
	PEOU3	0.797	0.871	
	PEOU4	0.823	0.861	
Attitudes towards the use of artificial intelligence	ATT1	0.727	0.866	0.887
	ATT2	0.714	0.870	
	ATT3	0.782	0.844	
	ATT4	0.796	0.839	
Student’s intrinsic motivation to learn AI	AIIM1	0.712	0.831	0.866
	AIIM2	0.695	0.838	
	AIIM3	0.722	0.827	
	AIIM4	0.735	0.821	
Artificial intelligence readiness	AIRD1	0.757	0.834	0.877
	AIRD2	0.783	0.823	
	AIRD3	0.689	0.860	
	AIRD4	0.712	0.851	
Artificial intelligence confidence	AICF1	0.814	0.890	0.916
	AICF2	0.814	0.889	
	AICF3	0.814	0.889	
	AICF4	0.791	0.898	
Artificial intelligence anxiety	AIAX1	0.769	0.857	0.891
	AIAX2	0.791	0.849	
	AIAX3	0.757	0.862	
	AIAX4	0.726	0.873	
User interface	UI1	0.685	0.860	0.864
	UI2	0.766	0.786	
	UI3	0.777	0.776	
Content	C1	0.790	0.842	0.888
	C2	0.745	0.860	
	C3	0.735	0.863	
	C4	0.750	0.858	
Learner-interface interactivity	LINT1	0.828	0.845	0.903
	LINT2	0.831	0.841	
	LINT3	0.772	0.893	

Secondly, KMO and Bartlett tests were conducted to analyze the effectiveness of the entire questionnaire. The results are shown in Table 5 below.

**Table 5:** KMO and Bartlett tests.

KMO value		0.898
Bartlett-test of sphericity	CMIN	5506.556
	DF	703
	P	0.000

From the Table 5, it can be seen that the KMO value is 0.880, and the KMO value is greater than 0.8, which illustrate the research data is very suitable for extracting information.

#### Discriminant validity

The results of the discriminant validity test are shown in Table 6. It can be seen that the AVE extracted square root (number on the diagonal) of each variable is greater than the correlation between this variable and other variables, so the data is considered to have good discriminant validity.

**Table 6:** Discriminant validity, Fornell–Larcker criterion.

Dimension	PU	PEOU	ATT	AIIM	AIRD	AICF	AIAX	UI	C	LINT
PU	0.860									
PEOU	0.690	0.881								
ATT	0.365	0.372	0.865							
AIIM	0.684	0.625	0.386	0.845						
AIRD	0.524	0.476	0.268	0.457	0.855					
AICF	0.556	0.534	0.304	0.474	0.311	0.894				
AIAX	− 0.449	− 0.435	− 0.268	− 0.411	− 0.248	− 0.228	0.868			
UI	0.286	0.447	0.403	0.301	0.235	0.354	− 0.198	0.887		
C	0.579	0.545	0.380	0.544	0.378	0.418	− 0.267	0.300	0.866	
LINT	0.535	0.526	0.315	0.502	0.320	0.387	− 0.251	0.239	0.284	0.916

According to Table 7, the above HTMT values are all below 0.85, indicating that the data has good discriminant validity.

**Table 7:** Discriminant validity: Heterotrait–Monotrait ratio (HTMT).

Dimension	PU	PEOU	ATT	AIIM	AIRD	AICF	AIAX	UI	C	LINT
PU										
PEOU	0.773									
ATT	0.412	0.412								
AIIM	0.781	0.703	0.435							
AIRD	0.595	0.535	0.302	0.522						
AICF	0.617	0.583	0.336	0.528	0.345					
AIAX	0.502	0.481	0.3	0.465	0.278	0.248				
UI	0.327	0.504	0.465	0.348	0.269	0.395	0.22			
C	0.654	0.609	0.428	0.617	0.429	0.464	0.298	0.343		
LINT	0.599	0.582	0.35	0.569	0.359	0.425	0.277	0.269	0.317	

### Structural Equation Evaluation

#### Model fitting index

The initial step in hypothesis testing involves assessing the structural model. Our model adheres to established fitting standards, with all model fitting index values deemed acceptable, including VIF < 5 and F2 > 0.02. Notably, all VIF values fall below 5, signifying the absence of significant collinearity concerns within the dataset.

#### Hypothesis testing

**Figure 4:**
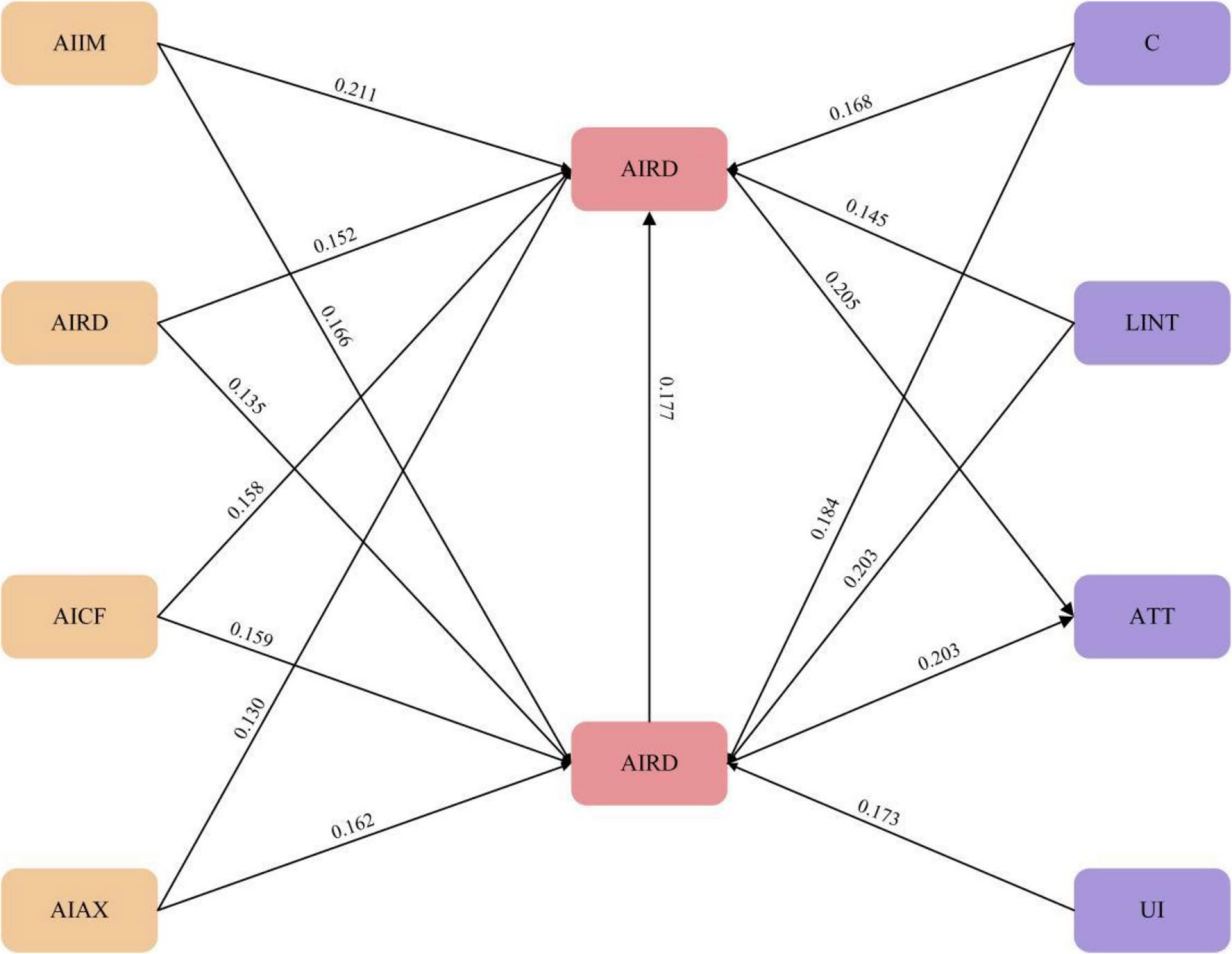
Results of hypothesis testing.

The sample size of this study is an important factor in model analysis. Therefore, after strict screening, 197 valid questionnaires were used for research analysis, which meets the sample size required for SMART PLS analysis. This study calculated the path coefficient and p-value. As shown in Fig. 4, the significance of all assumed pathways is supported at the 0.05 significance level.

The path coefficients of the structural equation model are shown in Table 8.

**Table 8:** Hypothesis test results of SEM.

Hypothesis	Path	β	P	Result
H1a	AIIM → PU	0.211	0.002	Support
H1b	AIIM → PEOU	0.166	0.011	Support
H2a	AIRD → PU	0.152	0.001	Support
H2b	AIRD → PEOU	0.136	0.011	Support
H3a	AICF → PU	0.158	0.002	Support
H3b	AICF → PEOU	0.159	0.004	Support
H4a	AIAX → PU	-0.130	0.009	Support
H4b	AIAX → PEOU	-0.162	0.001	Support
H5	UI → PEOU	0.173	0.000	Support
H6a	C → PU	0.168	0.003	Support
H6b	C → PEOU	0.184	0.002	Support
H7a	LINT → PU	0.145	0.003	Support
H7b	LINT → PEOU	0.203	0.000	Support
H8	PU → ATT	0.208	0.011	Support
H9	PEOU → PU	0.177	0.013	Support
H10	PEOU → ATT	0.228	0.027	Support

In this study, students’ cognitive factors such as AIIM, AIRD and AICF influence positively on PU. Hypothesis all have been tested as H1a (AIIM → PU, β = 0.211); H1b (AIIM → PEOU, β = 0.166), H2a (AIRD → PU, β = 0.152), H2b (AIRD → PEOU, β = 0.136), H3a (AICF → PU, β = 0.158), H3b (AICF → PEOU, β = 0.159), and p < 0.05. The indicates that AIIM, AIRD, and AICF have a positive impact on attitudes among cognitive factors in learning AIED through the use of GenAI. However, H4a (AIAX → PU, β =—0.130), H4b (AIAX → PEOU, β =—0.162), and p < 0.05, indicating that student AIAX has a negative impact on students’ ATT. Among the HCI factors, C and LINT have a positive impact on PU and PEOU, while UI has a positive impact on PEOU. After verification, these assumptions are valid and valid. While, H5 (UI → PEOU, β = 0.173), H6a (C → PU, β = 0.168), H6b (C → PEOU, β = 0.184), H7a (LINT → PU, β = 0.145), H7b (LINT → PEOU, β = 0.203), and p < 0.05. PU and PEOU have a positive impact on ATT, with path coefficients ranging from 0.17 to 0.23 with p < 0.05.

To sum up, PU, PEOU, AIIM, AIRD, AICF, UI, C, and LINT are important factors that positively affect students’ attitudes towards learning AIED through the use of GenAI, while AIAX has a negative impact on ATT.

#### The impact of AI learning cognitive factors on PU and PEOU

The results of the study showed that firstly, AIIM had a positive impact on PU and it was the second most influential factor (0.211) on student acceptance as well as positively affecting PEOU.

Although AIRD has a positive effect on PU (0.152) and PEOU (0.136), its positive impact on PEOU is indeed the smallest. This is consistent with the research of Chiu et al.^24^.which previously believed that the level of AIRD can measure the understanding of AI knowledge and technology.

AICF is positively correlated with PU (0.158) and PEOU (0.159), which is consistent with the results of graduate students using mobile devices for learning (Stavros A. Nikou, et al.). The greater the confidence in learning AI, the better students can accept AI courses and maintain sustainable learning behavior.

AIAX has a negative impact on both PU (− 0.130) and PEOU (− 0.162). This is consistent with the results of Tae Hyun Baek and Minseong Kim’s study on students’ learning behavior using ChatGPT^67^, and also with the results of Stavros A. Nikou et al.’s study on mobile device use anxiety.

#### Positive impact of HCI factors on PU and PEOU

Among the HCI factors, UI is positively correlated with PEOU, with a path coefficient of 0.173. UI is considered an important influencing factor in online course student acceptance (AL-Sayid, F. and Kirkil, G.)^29^ and mobile learning acceptance^77^. While using GenAI to teach courses, a more user-friendly interface makes it more likely for students to accept and choose this course.

In the results, C was found to be significantly positively correlated with PU (0.168) and PEOU (0.168).When using the GenAI tool for learning, LINT has a significant impact on PU and PEOU, with a path coefficient of 0.203.The use of programs through the menu bar to interact with the GenAI system has a significant impact on students’ acceptance.

#### Relationship between PU, PEOU, and ATT

We gained the following conclusion. PU has a positive impact on ATT (0.208). PEOU has a positive impact on PU (0.177). And PEOU has a positive impact on ATT (0.228). Previous research in the field of education has focused on the use of mobile devices and online courses. While the results of this study indicate that those factors are also applicable to the study of AI course acceptance by GenAI.

The results indicate that AIIM, AIRD, AICF, AIAX, UI, C and LINT all influence students’ attitudes towards learning AI. Among students' cognitive factors. AIIM has the greatest effect on PU, and among human HCI factors, LINT has the greatest effect on PEOU.

## Discussion

The discussion section of this study offers an in-depth analysis of K-12 students’ attitudes towards AI courses facilitated by generative AI (GenAI) tools. The examination is structured into three key segments, each focusing on distinct aspects of the research. Initially, the study investigates how cognitive factors related to AI learning influence perceived usefulness (PU) and perceived ease of use (PEOU). Subsequently, it explores the impact of human–computer interaction (HCI) factors on PU and PEOU. Finally, it delves into the interplay between PU, PEOU, and attitude towards use (ATT).Building upon established theoretical frameworks, such as the technology acceptance model (TAM), this study introduces a novel conceptual model tailored to assess K-12 students’ attitudes towards using GenAI tools in AI in education (AIED) courses. By incorporating both cognitive learning factors and HCI elements, the study extends the existing literature, offering a comprehensive understanding of the complex dynamics influencing students' attitudes towards AI education.

The empirical analysis conducted in this study validates the proposed model and hypotheses, thereby contributing to theoretical advancements in the field of AI4K12 education. However, it is crucial to contextualize these findings within the broader landscape of educational research. Previous studies, such as those by Almaiah and Almulhem (2018)^10^, Almaiah, Al-Khasawneh, and Althunibat (2020)^11^, and Almaiah and Al Mulhem (2020)^12^, have highlighted the critical challenges and success factors influencing the implementation and usage of e-learning systems. Drawing parallels between these studies and the current research can provide valuable insights into the unique considerations and obstacles associated with integrating innovative technologies, like GenAI, into educational settings.

From a practical perspective, the findings of this study underscore the potential of GenAI tools to enhance AIED methodologies. However, it is essential to recognize that variations in students’ cognitive learning processes may impact their attitudes and efficacy towards learning. By leveraging cutting-edge technologies and implementing pedagogical strategies informed by self-determination theory, educators and system designers can create inclusive and engaging learning experiences that promote sustained student engagement and mastery of AI knowledge.

In terms of theoretical implications, the findings of this study contribute significantly to the existing body of knowledge in the field of artificial intelligence in education (AIED). By expanding upon Davis’ technology acceptance model (TAM) with additional cognitive and human–computer interaction (HCI) factors, we have not only provided a more nuanced understanding of students’ attitudes towards AI courses facilitated by generative AI (GenAI) tools but also enriched the theoretical framework guiding research in this domain. This augmentation of the TAM model with external variables derived from the literature review and previous research findings offers a more comprehensive perspective on the determinants of students’ acceptance of AI4K12 courses. Furthermore, the empirical validation of this extended model through structural equation modeling adds robustness to its theoretical underpinnings and lays the groundwork for future research endeavors in the realm of AIED.

In conclusion, this study offers actionable insights for AIED policymakers, system developers, educators, and students, aiming to foster a superior AI learning experience for K-12 students. By addressing the complex interplay between cognitive factors, HCI elements, and attitudes towards AI education, this research contributes to the ongoing discourse surrounding the integration of GenAI tools in educational settings.

## Conclusion

The advent of artificial intelligence (AI) represents both significant opportunities and challenges for society, as intelligent algorithms and robots increasingly assume roles across various sectors. As AI becomes more integrated into daily life, it becomes crucial for individuals to adapt to coexist with these technologies. This underscores the importance of early integration of AI in education (AIED) into student learning, necessitating pioneering research in AIED-centric pedagogy for the K-12 demographic.

This study delves into K-12 students’ perceptions of learning AI-related content through generative AI (GenAI) tools. Through an extensive literature review, the study identifies external factors shaping students’ attitudes towards learning and applies the technology acceptance model (TAM), integrating it with theories of cognitive learning and human–computer interaction (HCI). With the participation of 210 Chinese K-12 students, this work stands as a significant contribution to the field. The analysis validates ten hypotheses, demonstrating the substantial impact of cognitive and behavioral learning factors, alongside HCI considerations, on students’ attitudes towards AI education. These findings offer crucial insights for AIED policymakers and developers, informing the creation of diverse and engaging AI4K12 curricula aimed at sustaining students’ interest in AI and promoting ongoing engagement and acquisition of intricate AI knowledge. However, this study has its limitations. The predominantly Chinese sample may not fully represent the global student body, and the study does not comprehensively cover all K-12 age groups. Future research should encompass a broader spectrum of K-12 grade levels, span multiple countries and regions, and explore gender and grade-level variations among students. Additionally, reliance on a single experimental course approach and online quantitative data collection may not fully capture the nuances of students’ attitudes. Future investigations should integrate qualitative methodologies, such as semi-structured interviews and group discussions, for deeper insights.

The results of this study underscore the importance of considering both cognitive learning factors and HCI elements in designing and implementing AI courses in K-12 education. Our findings suggest that enhancing students' perceptions of usefulness and ease of use, while addressing potential anxiety associated with AI, is crucial for fostering positive attitudes towards AI education. By integrating GenAI tools into the curriculum, educators can create more engaging and effective learning experiences for students, thereby promoting the development of essential AI literacy skills. Moreover, our study sheds light on the complex interplay between cognitive and HCI factors in shaping students' attitudes towards AI education, highlighting the need for a holistic approach to curriculum design. Furthermore, recent studies have contributed to the development of scales aimed at measuring artificial intelligence literacy and acceptance, providing valuable tools for researchers and educators to assess students' readiness and attitudes towards AI education^20–22^.

In conclusion, this research offers a thorough examination of K-12 students’ attitudes towards AI education using GenAI tools, focusing on learning cognition and HCI factors. Future endeavors should explore additional factors affecting AI learning acceptance, including various aspects of the learning environment, and examine students’ AI learning experiences from diverse cognitive viewpoints.

In addition to the research findings and limitations discussed above, it is essential to consider the practical and theoretical implications of this study. Practically, the findings offer valuable insights for educators, policymakers, and developers involved in AI education for K-12 students. By identifying the cognitive and HCI factors that influence students’ attitudes towards AI education using GenAI tools, this research provides a roadmap for designing more effective and engaging AI4K12 curricula. Educators can leverage these insights to tailor their teaching approaches and course designs to better meet students’ needs and preferences, ultimately fostering a more positive learning experience.

Moreover, policymakers can use this research to inform decisions regarding the integration of AI education into school curricula, ensuring that students are adequately prepared for the future workforce. From a theoretical perspective, this study contributes to the existing body of literature on AI education and technology acceptance by extending the TAM framework to include cognitive and HCI factors specific to GenAI tools. By validating the proposed model and hypotheses, this research advances our understanding of the complex interplay between individual perceptions, cognitive processes, and technological interfaces in the context of AI education. Furthermore, the inclusion of HCI factors underscores the importance of considering user experience and interface design in educational technology development, highlighting the need for a more holistic approach to AI education research. Overall, the practical and theoretical implications of this study underscore its significance and provide a foundation for future research in the field of AI education.

## Data Availability

The datasets generated and analyzed during the current study are available from the corresponding author upon reasonable request.

## Abbreviations

AI: Artificial intelligence
AIED: Artificial intelligence education
AI4K12: Artificial intelligence education for k-12
GenAI: Generative artificial intelligence
TAM: Technology acceptance mode
PU: Perceived usefulness
PEOU: Perceived ease of use
ATT: Attitudes towards the use of artificial intelligence
BI: Behavioral intent
SEM: Structural Equation model
AIIM: Student’s intrinsic motivation to learn artificial intelligence
AIRD: Artificial intelligence readiness
AICF: Artificial intelligence confidence
AIAX: Artificial intelligence anxiety
HCI: Human–computer interaction
UI: User interface
C: Content
LINT: Learner-interface interactivity
CT: Computational thinking
DT: Design thinking

## References

[1] Russell Stuart J, Norvig P (2010). Artificial intelligence a modern approach.

[2] OECD. Trustworthy Artificial Intelligence (AI) in Education, Promises and Challenges. https://www.oecd.org/education/trustworthy-artificial-intelligence-ai-in-education-a6c90fa9-en.html. Accessed 10 Oct 2023. (2020).

[3] Touretzky D, Gardner-Mccune C, Breazeal C, Martin F, Seehorn D (2019). A year in K-12 AI education. AI. Mag..

[4] Touretzky D, Gardner-McCune C, Martin F, Seehorn D. Envisioning AI for K-12, What should every child know about AI? In Proceedings of the AAAI conference on artificial intelligence, Honolulu, 17 July 2019; pp. 9795-9799. (2019).

[5] Ibe NA, Howsmon R, Penney L, Granor N, DeLyser LA, Wang K. Reflections of a diversity, equity, and inclusion working group based on data from a national CS education program. In *Proceedings of the 49th ACM Technical Symposium on Computer Science Education*. New York, NY, 21 February 2018; ACM, New York, NY, USA; pp. 711–716. (2018).

[6] Oermann EK, Kondziolka D (2022). On chatbots and generative artificial intelligence. Neurosurgery.

[7] Yu H, Guo Y (2023). Generative artificial intelligence empowers educational reform, current status, issues, and prospects. Front. Educ..

[8] Stokel-Walker C (2022). AI bot ChatGPT writes smart essays-should academics worry. Nature.

[9] Cooper G (2023). Examining science education in ChatGPT: An exploratory study of generative artificial intelligence. J. Sci. Educ. Technol..

[10] Almaiah MA, Almulhem A (2018). A conceptual framework for determining the success factors of e-learning system implementation using Delphi technique. J. Theor. Appl. Inf. Technol..

[11] Almaiah MA, Al-Khasawneh A, Althunibat A (2020). Exploring the critical challenges and factors influencing the E-learning system usage during COVID-19 pandemic. Educ. Inf. Technol..

[12] Almaiah M, Al Mulhem A (2020). Thematic analysis for classifying the main challenges and factors influencing the successful implementation of e-learning system using NVivo. Int. J. Adv. Trends Comput. Sci. Eng..

[13] Long D, Magerko B. What is AI Literacy? Competencies and Design Considerations. In *Proceedings of the 2020 CHI conference on human factors in computing systems*. New York, NY, USA, 23 April 2020, 1–16; ACM, New York, NY, USA; pp. 1–6. (2020).

[14] Zhou X, Van Brummelen J, Lin P (2020). Designing AI learning experiences for K-12: Emerging works, future opportunities and a design framework. Arxiv.

[15] Lin, P.; Van Brummelen, J. Engaging teachers to co-design integrated AI curriculum for K-12 classrooms. In *Proceedings of the 2021 CHI conference on human factors in computing systems*. Yokohama, Japan, 07 May 2021; ACM, New York, NY, USA; pp. 1–12. (2021).

[16] Sabuncuoglu A. Designing one year curriculum to teach artificial intelligence for middle school. In *Proceedings of the 2020 ACM conference on innovation and technology in computer science education*. New York, NY, USA, 15 June 2020; ACM, New York, NY, USA; pp. 96-102. (2020).

[17] Schleiss J, Laupichler MC, Raupach T, Stober S (2023). AI course design planning framework, developing domain-specific ai education courses. Educ. Sci..

[18] Ayanwale MA, Sanusi IT, Adelana OP, Aruleba KD, Oyelere SS (2022). Teachers’ readiness and intention to teach artificial intelligence in schools. Comput. Educ. Artif. Intell..

[19] Almaiah MA, Alfaisal R, Salloum SA, Hajjej F, Shishakly R, Lutfi A, Al-Maroof RS (2022). Measuring institutions’ adoption of artificial intelligence applications in online learning environments: Integrating the innovation diffusion theory with technology adoption rate. Electronics.

[20] Yilmaz R, Yilmaz FGK (2023). Augmented intelligence in programming learning: Examining student views on the use of ChatGPT for programming learning. Comput. Hum. Behav. Artif. Hum..

[21] Yilmaz FGK, Yilmaz R, Ceylan M (2023). Generative artificial intelligence acceptance scale: A validity and reliability study. Int. J. Hum. Comput. Interact..

[22] Yılmaz FG, Karaoğlan, and Ramazan Yılmaz,  (2023). Yapay Zekâ Okuryazarlığı Ölçeğinin Türkçeye Uyarlanması. Bilgi Ve İletişim Teknolojileri Dergisi.

[23] Terblanche N, Molyn J, Williams K, Maritz J (2023). Performance matters, students’ perceptions of artificial intelligence coach adoption factors. Coach. Int. J. Theor..

[24] Chai JL, Lin Y, Jong MSY, Dai Y, Chiu TK, Huang B (2020). Factors influencing students’ behavioral intention to continue artificial intelligence learning. International Symposium on Educational Technology (ISET).

[25] Zhou X, Van Brummelen J, Lin P. Designing AI learning experiences for K-12: Emerging works, future opportunities and a design framework. arXiv preprint arXiv:2009.10228. https://ar5iv.labs.arxiv.org/html/2009.10228. (2020).

[26] Wang N, Lester J (2023). K-12 education in the age of AI: A call to action for K-12 AI literacy. Int. J. Artif. Intell. Educ..

[27] Chiu TK, Meng H, Chai JL, King I, Wong S, Yam Y (2021). Creation and evaluation of a pretertiary artificial intelligence (AI) curriculum. IEEE Trans. Educ..

[28] Lv Z (2023). Generative artificial intelligence in the metaverse era. Cogn. Robot..

[29] Al-Sayid F, Kirkil G (2023). Exploring non-linear relationships between perceived interactivity or interface design and acceptance of collaborative web-based learning. Educ. Inf. Technol..

[30] Chen X, Xie H, Zou D, Hwang GJ (2020). Application and theory gaps during the rise of artificial intelligence in education. Comput. Educ. Artif. Intell..

[31] Shishakly R, Almaiah M, Lutfi A, Alrawad M (2024). The influence of using smart technologies for sustainable development in higher education institutions. Int. J. Data Netw. Sci..

[32] Holmes W, Bialik M, Fadel C (2023). Artificial Intelligence in Education.

[33] Ng DTK, Luo W, Chan HMY, Chu SKW (2022). Using digital story writing as a pedagogy to develop AI literacy among primary students. Comput. Educ. Artif. Intell..

[34] Dwivedi YK, Kshetri N, Hughes L, Slade EL, Jeyaraj A, KumarKar A, Baabdullah AM, Koohang A, Raghavan V, Ahuja M, Albanna H, Albashrawi MA, Al-Busaidi AS, Balakrishnan J, Barlette Y, Basu S, Bose I, Brooks L, Buhalis D, Carter L (2023). “So what if ChatGPT wrote it?” Multidisciplinary perspectives on opportunities, challenges and implications of generative conversational AI for research, practice and policy. Int. J. Inf. Manag..

[35] Chiu TK, Moorhouse BL, Chai JL, Ismailov M (2023). Teacher support and student motivation to learn with artificial intelligence (AI) based chatbot. Interact. Learn. Environ..

[36] Chiu TK (2023). The impact of generative AI (GenAI) on practices, policies and research direction in education: A case of ChatGPT and midjourney. Interact. Learn. Environ..

[37] Wu Y, Yu N, Li Z, Backes M, Zhang Y (2022). Membership inference attacks against text-to-image generation models. Arxiv.

[38] Watson AD, Watson GH (2013). Transitioning STEM to STEAM: Reformation of engineering education. J. Qual. Part..

[39] Cohn N (2020). Visual narrative comprehension: Universal or not. Psychon. B. Rev..

[40] Kim KH, Kim HG (2023). A study on how to create interactive children’s books using ChatGPT and midjourney. Techart J. Art Imaging Sci..

[41] Chocarro R, Cortiñas M, Marcos-Matás G (2023). Teachers’ attitudes towards chatbots in education, a technology acceptance model approach considering the effect of social language, bot proactiveness, and users’ characteristics. Educ. Stud..

[42] Roy R, Babakerkhell MD, Mukherjee S, Pal D, Funilkul S (2022). Evaluating the intention for the adoption of artificial intelligence-based robots in the university to educate the students. IEEE Access.

[43] Davis FD (1985). A Technology Acceptance Model for Empirically Testing New End-User Information Systems: Theory and Results.

[44] Davis FD (1989). Perceived usefulness, perceived ease of use, and user acceptance of information technology. Mis. Quart..

[45] Al-Adwan AS (2023). Extending the technology acceptance model (TAM) to Predict University students’ intentions to use metaverse-based learning platforms. Educ. Inf. Technol..

[46] Almaiah MA, Al-Otaibi S, Lutfi A, Almomani O, Awajan A, Alsaaidah A, Awad AB (2022). Employing the TAM model to investigate the readiness of M-learning system usage using SEM technique. Electronics.

[47] Almaiah MA, Ayouni S, Hajjej F, Lutfi A, Almomani O, Awad AB (2022). Smart mobile learning success model for higher educational institutions in the context of the COVID-19 pandemic. Electronics.

[48] Gursoy D, Chi OH, Lu L, Nunkoo R (2019). Consumers acceptance of artificially intelligent (AI) device use in service delivery. Int. J. Inform. Manage..

[49] Kelly S, Kaye SA, Oviedo-Trespalacios O (2022). What factors contribute to acceptance of artificial intelligence? A systematic review. . Telemat. Inform..

[50] Garrison DR, Anderson T, Archer W (2001). Critical thinking, cognitive presence, and computer conferencing in distance education. Am. J. Distance Educ..

[51] Chai JL, Wang X, Xu C (2020). An extended theory of planned behavior for the modelling of Chinese secondary school students’ intention to learn artificial intelligence. Mathematics.

[52] Lan YJ, Botha A, Shang J, Jong MSY (2018). Guest editorial: Technology enhanced contextual game-based language learning. J. Educ. Technol. Soc..

[53] Ryan RM, Deci EL (2000). Intrinsic and extrinsic motivations: Classic definitions and new directions. Contemp. Educ. Psychol..

[54] Froiland JM, Worrell FC (2016). Intrinsic motivation, learning goals, engagement, and achievement in a diverse high school. Psychol. Sch..

[55] Fagan MH, Neill S, Wooldridge BR (2008). Exploring the intention to use computers: An empirical investigation of the role of intrinsic motivation, extrinsic motivation, and perceived ease of use. J. Comput. Inform. Syst..

[56] Martín-Núñez JL, Ar AY, Fernández RP, Abbas A, Radovanović D (2023). Does intrinsic motivation mediate perceived artificial intelligence (AI) learning and computational thinking of students during the COVID-19 pandemic. Comput. Educ. Artif. Intell..

[57] Parasuraman A, Colby CL (2015). An updated and streamlined technology readiness index: TRI 2.0. J. Serv. Res..

[58] Dai Y, Chai JL, Lin PY, Jong MSY, Guo Y, Qin J (2020). Promoting students’ well-being by developing their readiness for the artificial intelligence age. Sustain. Sci..

[59] Ajzen I (1991). The theory of planned behavior. Organ. Behav. Hum. Decis..

[60] Lin PY, Chai JL, Jong MSY, Dai Y, Guo Y, Qin J (2021). Modeling the structural relationship among primary students’ motivation to learn artificial intelligence. Comput. Educ. Artif. Intell..

[61] Chai JL, Lin PY, Jong MSY, Dai Y, Chiu TK, Qin J (2021). Perceptions of and behavioral intentions towards learning artificial intelligence in primary school students. Educ. Technol. Soc..

[62] Owolabi K, Abayomi AO, Aderibigbe N, Kemdi OM, Oluwaseun OA, Okorie C (2022). Awareness and readiness of Nigerian polytechnic students towards adopting artificial intelligence in libraries. J. Inf. Knowl..

[63] Nikou SA, Economides AA (2017). Mobile-based assessment: Investigating the factors that influence behavioral intention to use. Comput. Educ..

[64] Ha JG, Page T, Thorsteinsson G (2011). A study on technophobia and mobile device design. Int. J. Contents.

[65] Johnson DG, Verdicchio M (2017). AI anxiety. J. Assoc. Inf. Sci. Tech..

[66] Wang YY, Wang YS (2022). Development and validation of an artificial intelligence anxiety scale: An initial application in predicting motivated learning behavior. Interact. Learn. Envir..

[67] Baek TH, Kim M (2023). Is ChatGPT scary good? How user motivations affect creepiness and trust in generative artificial intelligence. Telemat. Inform..

[68] Massey BL, Levy MR (1999). Interactivity, online journalism, and English-language web newspapers in Asia. J. Mass. Commun. Q..

[69] Mcmillan SJ (2005). The researchers and the concept: Moving beyond a blind examination of interactivity. J. Interact. Advert..

[70] Cho CH (2004). Effects of banner clicking and attitude toward the linked target ads on brand-attitude and purchase-intention changes. J. Glob. Acad. Market. Sci..

[71] Almaiah MA, Alfaisal R, Salloum SA, Hajjej F, Thabit S, El-Qirem FA, Al-Maroof RS (2022). Examining the impact of artificial intelligence and social and computer anxiety in e-learning settings: Students’ perceptions at the university level. Electronics.

[72] Head AJ (1999). Design Wise: A Guide for Evaluating the Interface Design of Information Resources.

[73] Cliff M, Dillon A, Richardson J (1996). User Centered Design of Hypertext and Hypermedia for Education.

[74] Wang SK, Yang C (2005). The interface design and the usability testing of a fossilization web-based learning environment. J. Sci. Educ. Technol..

[75] Lohr LL, Falvo DA, Hunt E, Johnson B, Khan BH (2007). Improving the usability of distance learning through template modification. Flexible Learning in an Information Society.

[76] Liu IF, Chen MC, Sun YS, Wible D, Kuo CH (2010). Extending the TAM model to explore the factors that affect intention to use an online learning community. Comput. Educ..

[77] Almaiah MA, Jalil MA, Man M (2016). Extending the TAM to examine the effects of quality features on mobile learning acceptance. J. Comput. Educ..

[78] Shee DY, Wang YS (2008). Multi-criteria evaluation of the web-based e-learning system, a methodology based on learner satisfaction and its applications. Comput. Educ..

[79] Terzis V, Economides AA (2011). The acceptance and use of computer based assessment. Comput. Educ..

[80] Lee BC, Yoon JO, Lee I (2009). Learners’ acceptance of e-learning in South Korea, theories and results. Comput. Educ..

[81] Isaias P, Issa T (2015). Sustainable design, HCI, usability and environmental concerns.

[82] Althunibat A, Almaiah MA, Altarawneh F (2021). Examining the factors influencing the mobile learning applications usage in higher education during the COVID-19 pandemic. Electronics.

[83] Al-Adwan AS (2024). Unlocking future learning: Exploring higher education students’ intention to adopt meta-education. Heliyon.

[84] Lee MC (2010). Explaining and predicting users’ continuance intention toward e-learning, an extension of the expectation-confirmation model. Comput. Educ..

[85] Duncan TG, Mckeachie WJ (2005). The making of the motivated strategies for learning questionnaire. Educ. Psychol..

[86] Chou C (2003). Interactivity and interactive functions in web-based learning systems, a technical framework for designers. Brit. J. Educ. Technol..

